# Anti-N-Methyl-D-Aspartate Receptor Encephalitis and Life-Threatening Sinus Node Dysfunction: A Case Report, Literature Review, and Analysis of 23 Cases

**DOI:** 10.7759/cureus.64472

**Published:** 2024-07-13

**Authors:** Aleksan Khachatryan, Vahagn Tamazyan, Margarita Sargsyan, Hakob Harutyunyan, Joel Alejandro, Ashot Batikyan

**Affiliations:** 1 Department of Internal Medicine, University of Maryland Medical Center, Midtown Campus, Baltimore, USA; 2 Department of Internal Medicine, Maimonides Medical Center, New York, USA; 3 Department of Cardiology, Heratsi Hospital Complex, Yerevan, ARM; 4 Department of Internal Medicine, North Central Bronx Hospital, New York, USA

**Keywords:** bradycardia, transvenous pacemaker, permanent pacemaker (ppm), asystolic cardiac arrest, autonomic dysfunction, teratoma, sinus arrest, sinus pause, sinus node dysfunction, anti nmda receptor encephalitis

## Abstract

Anti-N-methyl-D-aspartate receptor (anti-NMDAR) encephalitis is the most common form of autoimmune encephalitis, presenting with various psychiatric manifestations, including behavioral and cognitive impairments, movement disorders, decreased consciousness, dysphasia, seizures, and autonomic dysfunction. Autonomic dysfunction may involve hyperthermia, apnea, hypotension, tachycardia, and life-threatening manifestations of sinus node dysfunction (SND), such as bradycardia, sinus pause or arrest, and asystole. The severity and significance of SND are critical, as it is not uncommon for these patients to progress into asystolic cardiac arrest, potentially contributing to morbidity and mortality. Accordingly, we present the case of an 18-year-old female with anti-NMDAR encephalitis who experienced multiple episodes of sinus pause/arrest and asystolic cardiac arrest, achieving a return of spontaneous circulation after successful CPR in all instances, ultimately requiring permanent pacemaker implantation. Additionally, we performed a literature review and analyzed 23 similar anti-NMDAR encephalitis cases with SND manifestations, including sinus pause/arrest or asystolic cardiac arrest, to identify common risk factors and describe management strategies and outcomes. Moreover, we investigated the potential association between teratoma and permanent pacemaker use in SND.

## Introduction

Anti-N-methyl-D-aspartate receptor (anti-NMDAR) encephalitis is considered the most common form of noninfectious encephalitis, characterized by IgG antibodies against the NR1 subunit of the N-methyl-D-aspartate (NMDA) receptor. This condition is often associated with ovarian teratoma, and it is predominantly observed in females [1]. Clinical presentations are diverse, ranging from psychiatric disturbances, movement disorders, and seizures to autonomic dysfunction. Autonomic dysfunction can manifest as hypo/hyperthermia, hypoventilation, apnea, hypersalivation, dynamic ileus, hyperhidrosis, blood pressure lability, tachycardia, bradycardia, sinus pause/arrest, and asystolic cardiac arrest [1,2]. Tachycardia-bradycardia (tachy-brady), bradycardia, sinus pause/arrest, and asystolic cardiac arrest characterize sinus node dysfunction (SND) [3]. Specifically, sinus pause is defined as the absence of sinus node depolarization for more than three seconds, while sinus arrest involves the complete cessation of sinus depolarization that can progress into asystolic cardiac arrest in the absence of ectopic foci activation.

Accordingly, we present the case of an 18-year-old female diagnosed with anti-NMDAR encephalitis whose hospital course was complicated by tachy-brady syndrome and multiple episodes of sinus pause/arrest, progressing to cardiac arrest. The CPR efforts were successful in all episodes, resulting in the return of spontaneous circulation (ROSC). Ultimately, a permanent pacemaker was inserted.

Given the complexity of anti-NMDAR encephalitis and the need for respiratory or circulatory support and close monitoring of hemodynamic parameters in severe cases, most patients receive treatment in the intensive care setting [4]. SND and its most threatening complications, such as asystolic cardiac arrest and unstable bradycardia, add an extra layer of complexity and contribute to morbidity and mortality [1,5]. Recognizing the clinical significance and impact on poor outcomes, we performed a literature review to identify all cases of anti-NMDAR encephalitis with autonomic instability observed as asystolic cardiac arrest or sinus pause/arrest. We conducted extensive searches in PubMed, Google Scholar, and all other available sources published until March 2024, including only cases with a definitive diagnosis of anti-NMDAR encephalitis in the absence of alternative explanations for SND, such as electrolyte or metabolic abnormalities, medications, hypothyroidism, and so on. Given the younger median age of the study population, structural or myocardial infiltrative abnormalities were considered highly unlikely to be the underlying causes of SND. The search terms used for this review included "Anti-N-methyl-D-aspartate receptor encephalitis and cardiac arrest," "Anti-N-methyl-D-aspartate receptor encephalitis and sinus node dysfunction," "Anti-N-methyl-D-aspartate receptor encephalitis and sinus arrest/pause," and "Anti-N-methyl-D-aspartate receptor encephalitis and asystole." Moreover, we included only cases with sufficient information regarding patients' demographics, encephalitis treatment, diagnostic workup, treatment of sinus pause/arrest, cardiac arrest, and outcomes.

The objective of analyzing all similar cases was to identify the risk factors, describe management strategies, and evaluate the association between pacemaker utilization and patients' outcomes. Additionally, we hypothesized that patients with teratoma would have fewer permanent pacemaker implantations than those without teratoma, given the data suggesting that teratoma is associated with full recovery and better outcomes [1,6].

## Case presentation

An 18-year-old female with a medical history of posttraumatic stress disorder (PTSD), obesity, polycystic ovary syndrome (PCOS), hypertension, migraine, and psychogenic non-epileptic seizures (PNES) was admitted to the intensive care unit (ICU) because of seizure-like activity leading to hypoxia.

Before ICU admission, the patient had been receiving treatment in the psychiatric unit for complex PTSD characterized by disorganization, bizarre behavior, agitation, aggression, and delusions. Over the past two months, she had experienced a deterioration of these symptoms. Her treatment involved lurasidone, escitalopram, and amlodipine. The family history was noncontributory. The patient had no history of illicit drug use.

The vital signs were as follows: temperature 36.6°C, heart rate 123 beats per minute, blood pressure 128/60 mmHg, respiratory rate 32 breaths per minute, and oxygen saturation 94% on high-flow nasal cannula. The patient did not appear to be in distress and was slightly somnolent. Physical examination revealed tachycardia and diminished bilateral breathing sounds in the basal fields of the lungs. The neurological examination was unremarkable without localizing findings.

**Figure 1:**
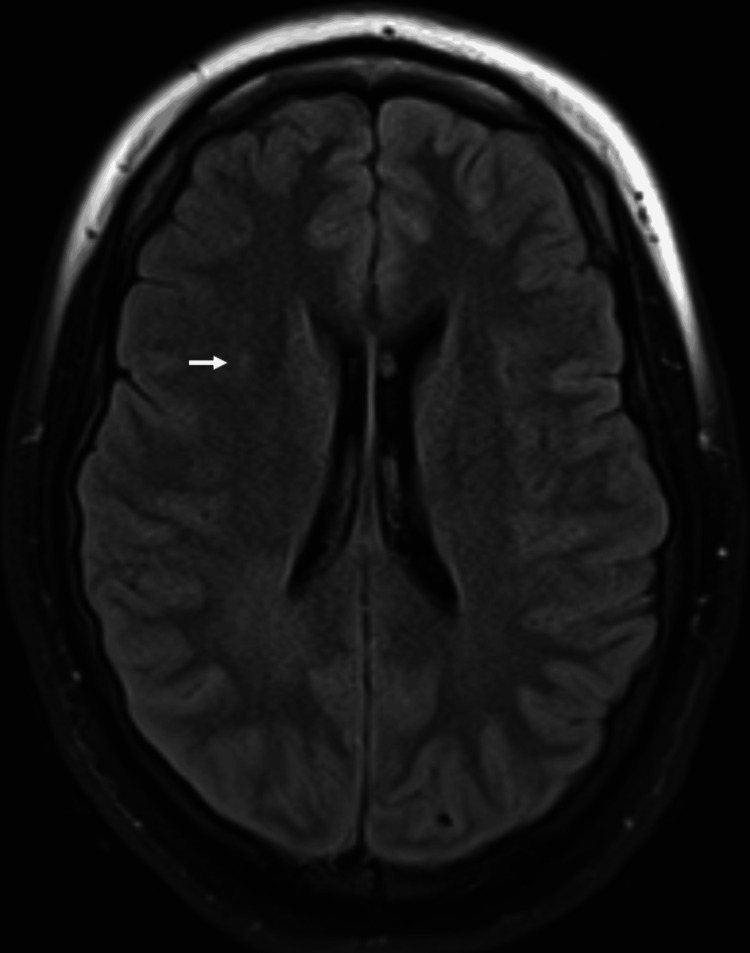
Brain MRI A brain MRI reveals a subtle increased FLAIR signal in the right periventricular white matter, as indicated by the white arrow. FLAIR: fluid-attenuated inversion recovery

While in the ICU, the patient experienced several other seizure-like episodes characterized by upper extremity extension and facial twitching accompanied by changes in mental status, hypoxia, tachypnea, and tachycardia. These episodes were different from prior events. Laboratory workup revealed an elevated lactate level of 4.9 mmol/L (reference range 0.7-2.0 mmol/L). Continuous electroencephalography (cEEG) eventually confirmed generalized tonic-clonic seizures originating from the right frontal lobe. Subsequently, a loading dose of midazolam was given, and the patient was started on lacosamide and valproate. Brain MRI revealed right periventricular fluid-attenuated inversion recovery (FLAIR) changes, which were considered to be the sequelae of multiple seizure activities (Figure 1). Several attempts at lumbar puncture (LP) were unsuccessful because of the patient's body habitus.

The hypoxia was attributed to aspiration during the seizures. It should be noted that the patient was consistently tachycardic for approximately 17 days following the diagnosis of seizures, with a heart rate ranging from 100 to 120 beats per minute. An ECG demonstrated sinus tachycardia. The CT angiography (CTA) of the chest was negative for pulmonary embolism. Thyroid-stimulating hormone (TSH) levels were within the normal limits of 1.29 mIU/L (reference range 0.47-4.68 mIU/L).

The patient remained stable on lacosamide and valproate without any new clinical or EEG-based seizure episodes. Propranolol was also added to the treatment regimen to address physical aggressiveness. Because of the inability to obtain CSF samples, a blood workup for an autoimmune encephalitis panel was ordered.

On the 17th day after the diagnosis of seizures, the serum anti-NMDAR antibody titer was positive at 1:320. The rest of the encephalitis panel, including anti-glutamic acid decarboxylase (anti-GAD), aquaporin-4 receptor (NMO/AQP4), dipeptidyl-peptidase-like protein 6 (DPPX), α-amino-3-hydroxy-5-methyl-4-isoxazolepropionic acid receptor (AMPAR), contactin-associated protein-2 (CASPR2), gamma-aminobutyric acid receptor type A (GABA-AR), gamma-aminobutyric acid receptor type B (GABA-BR), leucine-rich glioma-inactivated protein 1 (LGI1), metabotropic glutamate receptor 1 (mGluR1), and IgLON family member 5 (IgLON5) antibodies, were negative.

Additional workups, including serum human immunodeficiency virus (HIV), rapid plasma reagin (RPR) titers, QuantiFERON®-TB Gold blood test, acute hepatitis panel, anti-Borrelia burgdorferi antibodies, and urine toxicology, were negative. The levels of vitamin B12, folate, antinuclear antibody (ANA), Scl-70 antibody, SSA 52 (Ro) (ENA) antibody, SSA 60 (Ro) (ENA) antibody, SSB (La) (ENA) antibody, alpha-fetoprotein (AFP), tumor markers CA 125 and CA 19-9 were also within normal limits.

The management involved methylprednisolone 1 g IV for five days and intravenous immunoglobulin (IVIG) 2 g for three days. On the second day of immunotherapy, an episode of vomiting, aspiration, hypoxia, and bradycardia progressing to asystole was observed. ROSC was achieved two minutes after initiating CPR, and the patient was subsequently intubated for airway protection. Propranolol and lacosamide were discontinued, and lurasidone was tapered off. While receiving dexmedetomidine, the patient experienced bradycardia with heart rates of 49-50 beats per minute and episodes of sinus pauses lasting approximately three to four seconds, often triggered by coughing spells. Accordingly, dexmedetomidine was also discontinued. Albuterol was initiated for the persistent bradycardia. A few days after discontinuing lacosamide, propranolol, and dexmedetomidine, a sinus pause lasting approximately forty-five seconds during repositioning on her left side was recorded on telemetry. This episode self-resolved just before the initiation of chest compressions. The ECG related to this event was unremarkable. Subsequently, epinephrine IV was initiated for the persistent bradycardia. Another episode of a sinus pause lasting approximately 45 seconds occurred after the discontinuation of epinephrine, which responded well to atropine administration. Several other episodes of bradycardia, with heart rates dropping to 30 beats per minute, were observed in association with endotracheal tube suctioning.

The patient underwent a successful LP eight days after the diagnosis of anti-NMDAR encephalitis and the completion of steroids and IVIG. The CSF findings are presented in Table 1.

**Table 1:** CSF findings NMDAR: N-methyl-D-aspartate receptor; Ab: antibody

Laboratory parameter	Results	Reference range
Glucose	56	40-70 mg/dL
Protein	14	12-60 mg/dL
RBC	1	0-0/mcL
WBC	6	0-5/mcL
Polys (%)	0	%
Lymphs (%)	98	%
Monos (%)	2	%
Color/clarity	Colorless/clear	Colorless/clear
CSF electrophoresis	No oligoclonal bands were detected	No oligoclonal bands were detected
CSF immunofixation	No oligoclonal bands were detected	No oligoclonal bands were detected
NMDAR Ab	1:20	<1:1

A transvenous pacemaker (TVP) was placed on the eighth day following the diagnosis of anti-NMDAR encephalitis. The TVP was inadvertently removed three days later, though no sinus pauses were reported during this period. Subsequently, the heart rate became tachycardic, and the sinus tachycardia persisted even after the discontinuation of albuterol.

Importantly, the workup for teratoma was conducted alongside the treatment of anti-NMDAR encephalitis. Initially, a CT scan of the abdomen and pelvis was concerning for an ovarian cyst. However, transabdominal and transvaginal ultrasound findings were supportive of PCOS. The pelvic MRI also demonstrated features consistent with PCOS, without evidence of a teratoma. Furthermore, a whole-body positron emission tomography scan was negative for teratoma or other malignancies and did not indicate any underlying inflammatory process in the myocardium.

**Figure 2:**
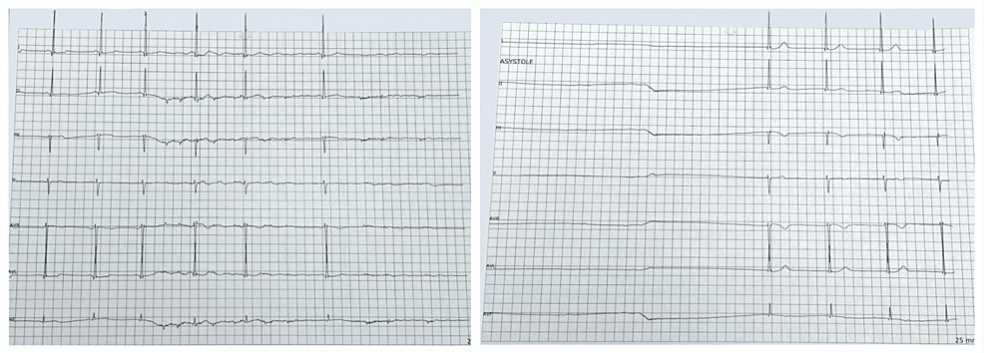
Telemonitoring This figure illustrates the onset (left panel) and termination (right panel) of a sinus pause, captured from telemonitoring. The ECG leads used in this recording, listed from top to bottom, are I, II, III, V1, aVR, aVL, and aVF.

The hospital course was notable for another cardiac arrest seventeen days after the diagnosis of anti-NMDAR encephalitis while being treated for a urinary tract infection (UTI) and bacteremia. Initially, a sinus pause was observed lasting approximately twenty seconds, which resolved spontaneously (Figure 2). Dexmedetomidine was discontinued, but five hours later, the patient had another episode of sinus pause precipitated by a cough, progressing to asystolic cardiac arrest. ROSC was achieved after two minutes of CPR. ECGs taken before and immediately after the cardiac arrest were unremarkable and showed sinus rhythm.

Subsequently, the cardiac MRI demonstrated normal biventricular size and function, with no evidence of focal abnormal late gadolinium enhancement to suggest fibrosis, scarring, or an infiltrative process. The underlying etiology of the cardiac arrest was attributed to severe dysautonomia in the context of anti-NMDAR encephalitis. Consequently, the patient underwent a successful single-chamber permanent pacemaker placement. Following this, an attempt to change the lower rate limit of the pacemaker to 30 beats per minute recorded several episodes of pacemaker activation.

The treatment with steroids and IVIG did not result in significant clinical improvement. Because of the prolonged course, tracheostomy and percutaneous endoscopic gastrostomy (PEG) tubes were placed. Following the treatment of the UTI and bacteremia, the patient was started on rituximab and a prednisone taper, but there was no significant improvement in mental status. Given the poor neurological recovery, the patient is currently receiving monthly cyclophosphamide at a rehabilitation facility.

## Discussion

The patient presented with behavioral and cognitive changes accompanied by seizures. Initial blood tests revealed positive anti-NMDAR titers, consistent with a diagnosis of anti-NMDAR encephalitis. Notably, the workup for teratoma was negative. The diagnosis was further confirmed by CSF analysis, which showed positive anti-NMDAR titers at 1:20. It is important to note that the patient had completed first-line immunotherapy before CSF sampling, which could potentially improve antibody titers.

The patient had multiple episodes of sinus pauses and arrests triggered by vomiting, coughing, suctioning, and repositioning, suggesting the role of vagotonia in these events. These episodes persisted even after the discontinuation of negative chronotropic agents such as propranolol, dexmedetomidine, and lacosamide, highlighting the link between anti-NMDAR encephalitis and autonomic dysfunction. Following the diagnosis of seizures, the patient remained tachycardic until the first cardiac arrest. The postintubation period was characterized by multiple sinus pauses and bradycardia for about a week, necessitating an epinephrine drip until a temporary pacemaker was inserted. The temporary pacemaker recordings were benign, with no bradycardic events, sinus pauses, or arrests. Interestingly, when the pacemaker was inadvertently removed, the patient had tachycardia, suggesting that reinsertion was not immediately necessary. However, seventeen days after the initial diagnosis, the patient experienced another asystolic cardiac arrest, leading to the insertion of a permanent pacemaker. This indicates the persistence of SND despite uneventful periods recorded on the temporary pacemaker. This case highlights the potential need for a longer duration of pacemaker use during the initial stages of anti-NMDAR encephalitis in patients with severe disease.

Our search identified twenty-two cases that met the specified criteria (see Table 2 for details) [7-25].

**Table 2:** Characteristics of the cases TTE: transthoracic echocardiography; tachy-brady: tachycardia-bradycardia; TCP: transcutaneous pacemaker; TVP: transvenous pacemaker; T: tracheostomy; P: percutaneous gastrostomy tube; N/A: not available; B/L: bilateral; R: right; L: left; IVIG: intravenous immunoglobulin; WNL: within normal limits; EF: ejection fraction; LVH: left ventricular hypertrophy; F: female; M: male

Author	Journal	Year	Age (years)	Sex	Tumor (any)	TTE	Brain imaging	Triggers	Cardiac arrest, sinus pause or arrest, bradycardia, tachy-brady	TCP or TVP	Permanent pacemaker	Encephalitis treatment	Tumor removal	T or P	Outcomes
Nazif et al. [7]	Europace	2012	18	F	B/L ovarian teratoma	EF 55%, WNL	B/L medial temporal extra-temporal	Cough, gag reflex, tracheostomy manipulation, bronchoscopy	Asystole, sinus arrest, tachy-brady	TVP	No	Steroids, plasmapheresis, IVIG, rituximab	B/L ovarian cystectomy	T, P	Complete recovery
Nazif et al. [7]	Europace	2012	19	F	R ovarian teratoma	EF 60%, WNL	B/L medial temporal	Nasogastric tube placement	Asystole, sinus arrest, tachy-brady	None	No	Steroids, IVIG	R salpingo-oophorectomy	P	Complete revovery
Nazif et al. [7]	Europace	2012	20	F	L ovarian teratoma	EF 60%, WNL	B/L medial temporal	Cough, bowel movements	Asystole, sinus arrest, tachy-brady	TCP	No	Plasmapheresis, IVIG, rituximab	L ovarian cystectomy	T, P	Recovery with mild residual deficit
Nazif et al. [7]	Europace	2012	33	F	None	EF 60%, mild LVH	N/A	Cough, suctioning	Asystole, sinus arrest, tachy-brady	TCP	No	Diagnosed retrospectively and lost to follow-up	None	T, P	Poor recovery
Patel et al. [8]	Am J Med Case Rep	2020	36	F	L ovarian teratoma	N/A	WNL	Related and unrelated to vagal stimuli	Sinus arrest, tachy-brady	TVP	No	Steroids, plasmapheresis, IVIG, rituximab	B/L oophorectomy	N/A	Poor recovery
Patel et al. [9]	Am J Respir Crit Care Med	2019	21	F	None	N/A	WNL	N/A	Asystole, bradycardia	TVP	No	Plasmapheresis, steroids, IVIG, rituximab	None	None	N/A
Mehr et al. [10]	Case Rep Neurol Med	2016	31	F	None	N/A	N/A	Coughing, suctioning of the endotracheal tube, and defecation	Asystole, sinus arrest, tachy-brady	TCP (required permanent pacemaker)	Yes	Steroids, IVIG, rituximab, intrathecal methotrexate	None	None	Complete recovery
Sansing et al. [11]	Nat Clin Pract Neurol	2007	34	F	L ovarian teratoma	WNL	B/L medial temporal lobe, predominantly left hippocampus	N/A	Asystole, bradycardia	None	No	Plasmapheresis, steroids, IVIG, cyclophosphamide	L salpingo-oophorectomy	T, P	Complete recovery
Day et al. [12]	J Gen Intern Med	2011	21	F	R ovarian teratoma	N/A	WNL	N/A	Cardiac arrest, third-degree AV block	TVP	No	Plasmapheresis, IVIG	R oophorectomy	N/A	Deceased
Shah et al. [13]	J Am Coll Cardiol	2016	37	F	B/L ovarian teratomas	N/A	N/A	N/A	Asystole, tachy-brady	TVP	No	Plasmapheresis	B/L oophorectomy	N/A	N/A
Millichap et al. [14]	Pediatrics	2011	15	F	Ovarian teratoma	WNL	L posterior temporal lobe	Seizure	Asystole, bradycardia	TVP (required permanent pacemaker)	Yes	Steroids, IVIG	Resection of ovarian teratoma	None	Complete recovery
Haththotuwa et al. [15]	JICS	2012	19	F	None	N/A	WNL	N/A	Asystole, tachy-brady	None	No	Steroids, IVIG	None	T	Recovery with mild residual deficit
Nazi et al. [16]	PJNS	2016	66	M	None	WNL	N/A	N/A	Asystole, bradycardia	None	No	Steroids, plasmapheresis	None	N/A	Deceased
Valente et al. [17]	Eur J Case Rep Intern Med	2023	25	M	None	N/A	WNL	N/A	Asystole, bradycardia	None	No	Steroids, IVIG, rituximab	None	N/A	Recovery with mild residual deficit
Wójtowicz et al. [18]	Anaesthesiol Intensive Ther	2017	23	F	L ovarian teratoma	N/A	WNL	Manipulations within the tracheostomy tube	Asystole, tachy- brady	None	No	Steroids, IVIG, plasmapheresis, cyclophosphamide, rituximab	L ovarian teratoma resection	T, P	Complete recovery
Kümpfel et al. [19]	Nat Clin Pract Neurol	2016	15	F	Thyroid gland teratoma	N/A	WNL	N/A	Asystole, bradycardia	TVP	No	Steroids, IVIG, plasmapheresis, rituximab	Thyroid gland teratoma removal	N/A	Complete recovery
Athar et al. [20]	Heart rhythm	2021	36	F	Ovarian teratoma	WNL	N/A	N/A	Cardiac arrest, sinus pause	TVP (required permanent pacemaker)	Yes	Immunotherapy	Ovarian tumor resection	N/A	N/A
Chawla et al. [21]	Int J Cardiol	2016	29	F	L ovarian teratoma	N/A	N/A	N/A	Asystole, sinus arrest, tachy-brady	TVP	No	Steroids, IVIG, plasmapheresis, rituximab	Left ovarian teratoma resection	N/A	Complete recovery
Lee et al. [22]	J Clin Neurosci	2011	41	F	None	WNL	L mesial temporal	Seizure, postictal period	Asystole, bradycardia	TVP (required permanent pacemaker)	Yes	Steroids	None	N/A	Complete recovery
Salehi et al. [23]	Am J Case Rep	2018	21	F	R ovarian teratoma	N/A	R medio-temporal lobe	N/A	Sinus pause, bradycardia	TVP (required permanent pacemaker)	Yes	Steroids, IVIG plasmapheresis	B/L salpingoophorectomy	T, P	Poor recovery
Wong et al. [24]	Acta Cardiol Sin	2023	29	F	None	WNL	WNL	Seizures	Asystole, sinus arrest, tachy-brady	TVP	No	Steroids, plasmapheresis, rituximab	None	None	Complete recovery
Ziaeian et al. [25]	Tex Heart Inst J	2015	19	F	R ovarian teratoma	N/A	WNL	N/A	Asystole, sinus pause, tachy-brady	TVP	No	Steroids, plasmapheresis	Right ovarian cystectomy	None	Complete recovery
Our case	Cureus	2024	18	F	None	WNL	R periventricular white matter	Cough, suctioning, vomiting	Cardiac arrest, sinus arrest, tachy-brady	TVP (required permanent pacemaker)	Yes	Steroids, IVIG, rituximab, cyclophosphamide	None	T, P	Poor recovery

The analysis of the case reports, including our case and the outcomes of different variables, is summarized in Table 3.

**Table 3:** Analysis of different variables of the reviewed cases Patients who received permanent pacemaker implantation were classified as not having TCP or TVP as their definitive treatment. For the analysis of outcomes, cases with no available information were also included. Tachy-brady: tachycardia-bradycardia; TCP: transcutaneous pacemaker; TVP: transvenous pacemaker

Variable	Category	Total (N=23)
Sex	Female	21 (91.3%)
	Male	2 (8.7%)
Arrhythmia	Other	2 (8.7%)
	Bradycardia	8 (34.8%)
	Tachy-brady	13 (56.5%)
TCP or TVP	No	12 (52.2%)
	Yes	11 (47.8%)
Permanent pacemaker	No	17 (73.9%)
	Yes	6 (26.1%)
Outcomes	Complete neurological recovery or recovery with mild residual deficit	14 (60.9%)
	Poor neurological recovery or death	6 (26.1%)
Teratoma	No	9 (39.1%)
	Yes	14 (60.9%)

The median age of the patients was twenty-three years, ranging from fifteen to sixty-six years. The female-to-male ratio was 10:1. Teratoma was more prevalent in this population (60.9%) than in the general anti-NMDAR encephalitis population, where it is observed in 38% of cases [26]. Complete neurological recovery or recovery with mild residual deficits was observed in approximately 60.9% of the patients, which is lower than the 81% recovery rate of the general anti-NMDAR encephalitis population [27].

All available transthoracic echocardiograms were unremarkable. Among identified cases, tachy-brady was reported in 56.5% of cases, while bradycardia occurred in 34.8% of cases. Regardless of tachy-brady or bradycardia, the patients experienced sinus pause/arrest or asystolic cardiac arrest at some point. Nearly all patients received some form of immunotherapy, including steroids, IVIG, plasmapheresis, rituximab, or cyclophosphamide. The most common triggers for bradycardic and asystolic events were cough, manipulations on the tracheostomy, and seizures.

Temporary TVP or transcutaneous pacemaker (TCP) was used as a definitive treatment in 47.8% of cases, while 26.1% of subjects required a permanent pacemaker. Although one review reported no permanent pacemaker use among ten patients, our findings suggest that autonomic dysfunction is not universally transient and may persist in some cases [7]. As illustrated in Table 4, the odds ratio of permanent pacemaker use in patients with teratoma is 0.56, which is not statistically significant, possibly because of the small sample size. However, it may potentially indicate that these patients are less likely to require a permanent pacemaker.

**Table 4:** Odds ratio calculation for the association between teratoma and permanent pacemaker use The odds ratio, calculated using Fisher's exact test, is 0.56 (95% CI: 0.06 to 5.56). As the CI includes 1, the odds ratio is not statistically significant (p = 0.643).

Variable	Level	Permanent pacemaker: Yes (N=6)	Permanent pacemaker: No (N=17)	Odds ratio
Teratoma	Yes	3 (50%)	11 (64.7%)	0.56 (CI, 0.06-5.56), P=0.643
	No	3 (50%)	6 (35.3%)	

Interestingly, only 40% (two out of five) of patients with permanent pacemakers demonstrated poor neurological recovery. As shown in Table 5, the use of a permanent pacemaker was associated with a statistically nonsignificant 78% higher odds of poor neurological recovery or death.

**Table 5:** Odds ratio calculation for the association between permanent pacemaker use and outcomes The odds ratio based on Fisher's exact test is 1.78 (95% CI: 0.11 to 22.86). Since the CI includes 1, the odds ratio is not statistically significant (p = 0.6126).

Variable	Level	Poor recovery or death (N=6)	Complete neurological recovery or recovery with a mild residual deficit (N=14)	Odds ratio
Permanent pacemaker	Yes	2 (33.3%)	3 (21.4%)	1.78 (CI, 0.11-22.86), P=0.6126
	No	4 (66.7%)	11 (78.6%)	

In Tables 4-5, we used Fisher's exact test to calculate the odds ratio given the small sample size. It should be noted that the odds ratio did not achieve statistical significance in either instance.

History and epidemiology

Anti-NMDAR encephalitis represents an autoimmune neurological disorder, first characterized in detail by Dalmau et al. in 2007, and has since emerged as an essential consideration in differential diagnoses for young individuals, particularly women, presenting with acute neuropsychiatric symptoms, autonomic instability, and cardiac dysrhythmias [28]. In another study conducted by Dalmau et al., it was found that among patients with anti-NMDAR encephalitis, 69 exhibited autonomic instability, and 37 experienced cardiac dysrhythmias such as tachycardia or bradycardia, with prolonged pauses observed in seven patients, four of whom required pacemaker implantation [27]. The association of this condition with ovarian teratomas in approximately half of the cases further complicates its clinical landscape, intertwining it with oncological considerations [27,28]. Initially identified as a paraneoplastic syndrome, anti-NMDAR encephalitis is now recognized to affect a broader demographic, including children and males, beyond its initial association. It is considered an uncommon autoimmune disorder, with an estimated yearly occurrence of one affected out of 1.5 million people [29]. It is more frequently observed in females, with the ratio of affected women to men being about 4:1. Further reports identified a median patient age of 21, ranging from infants to 85 years [26].

Pathogenesis

The central heart rate control is complex. The resting heart rate is regulated by the interaction of centrally mediated sympathetic and parasympathetic discharges and the cardiac pacemaker. Anti-NMDAR encephalitis is characterized by the production of autoantibodies against the NR1 and NR2 subunits of the NMDA receptor, which are essential for synaptic transmission [1]. The binding of these autoantibodies leads to receptor downregulation and internalization, including in the autonomic centers of the brain, disrupting glutamatergic neurotransmission and resulting in a range of neuropsychiatric symptoms, as well as dysregulation of heart rate, blood pressure, and temperature [1,30]. Specifically, the hypothalamus and brainstem, critical regions responsible for autonomic control, are affected by the antibody-mediated disruption of NMDA receptors [4].

Autonomic dysregulation in anti-NMDAR encephalitis is attributed to several mechanisms. First, the lock-step phenomenon (LSP) describes the simultaneous activation of sympathetic and parasympathetic discharges during epileptiform activity, culminating in fatal bradyarrhythmia, tachyarrhythmia, and sinus pause or arrest [31-33]. Second, as discussed above, the downregulation of NMDA receptors via antibody binding and internalization occurs primarily in the telencephalon, anterior cingulate cortices, insula, and amygdala. This leads to disruption of vagal stimuli to the heart, resulting in inappropriate sinus tachycardia [34]. As a result, the compensatory upregulation of secondary pathways that respond to afferent vagal stimuli leads to inappropriate bradycardia and sinus arrest. Finally, Cushing's triad, resulting from elevated intracranial pressure, manifests as widened pulse pressures, bradycardia, and irregular respirations, contributing to the complex dysrhythmias observed in anti-NMDAR encephalitis. Elucidating these mechanisms is essential for developing effective therapeutic strategies [8].

Clinical presentation

Patients typically present with various symptoms that initially mimic psychiatric conditions, including hallucinations and agitation, progressing to seizures, movement disorders, and autonomic instability, including cardiac dysrhythmias such as tachycardia, bradycardia, and asystole. This wide clinical spectrum determines morbidity and mortality, necessitating early recognition and management [35]. Bradyarrhythmias and asystolic events are most commonly triggered by vagal maneuvers such as coughing, defecation, and tracheal manipulations, as demonstrated in our analysis. Another common trigger for asystolic and bradycardic events is seizure or epileptic activity. The manifestations of SND depend on the duration of the sinus pause or the degree of bradycardia. In severe cases, patients may become hemodynamically unstable, showing signs of hypoperfusion and sometimes requiring CPR.

Diagnosis

The diagnosis integrates clinical presentation with the detection of anti-NMDAR antibodies in CSF and serum, supported by findings on MRI, such as signal hyperintensity in the hippocampus, and EEG, such as extreme delta brushes [36]. The diagnosis can be challenging because of overlap with other conditions, emphasizing the need for comprehensive evaluation and consideration of anti-NMDAR encephalitis in differential diagnoses of acute neuropsychiatric syndromes [35]. Given the complexity of the disease and the need for prolonged hospitalization, the most common diagnostic tool for assessing SND manifestations is potentially the analysis of cardiac telemetry recordings in hospital settings [4].

Treatment

Treatment primarily involves immunotherapy and, when applicable, tumor removal. First-line immunotherapies include corticosteroids, IVIG, and plasmapheresis, while rituximab and cyclophosphamide are second-line therapies for refractory cases [1]. Surgical removal of an associated ovarian teratoma or other neoplasms is crucial. Despite the disease's severity, prompt and aggressive treatment can significantly enhance recovery, emphasizing the importance of timely intervention [35,26].

The treatment modalities for sick sinus syndrome (SND) do not differ from generally accepted approaches. In acute settings, pharmacologic interventions such as atropine, dopamine, and epinephrine should be initiated promptly, followed by either TCP or TVP implantation if necessary [37]. Our analysis indicates that 47% of patients required temporary pacing (TCP or TVP) because of the reversible nature of the disease. It is a well-validated practice to treat symptomatic sinus pause or arrest with temporary pacing if the etiology is reversible. However, if the disease process involving the sinus node is nonreversible, the implantation of a permanent pacemaker is appropriate [3]. The majority of anti-NMDAR encephalitis patients (53%) demonstrate neuropsychiatric improvement within the first four weeks of therapy initiation, but it can extend up to 24 months. Approximately 81% of patients achieve substantial neurological recovery within 24 months [26]. However, there are no data about the duration of autonomic dysfunction. Accordingly, the exact duration of temporary pacemaker use remains unknown. The fact that around 19% of patients demonstrate poor neurological recovery suggests an irreversible nature of the disease in these cases. Whether this irreversibility involves the autonomic regulation centers of the brain, posing a permanent risk of SND with potentially fatal outcomes, is still unclear. As we stated earlier, 26% of our patients required permanent pacemakers, mostly secondary to the protracted course of SND, possibly indicating the irreversible nature of central autonomic dysregulation.

Notably, not all patients requiring permanent pacemakers necessarily had poor neurological recovery or fatal outcomes, although the odds of poor neurological outcomes or death were 78% higher in patients requiring permanent pacemakers. Therefore, the timeline for temporary pacemaker removal should be individualized, considering both the patient's overall neurological status and, most importantly, the pacemaker's interpretation data to ensure the absence of life-threatening bradyarrhythmia and asystole before removal.

The association between autonomic instability, particularly cardiac arrhythmias, and anti-NMDAR encephalitis underscores the critical importance of this autoimmune neurological disorder. It is important to note the limitations of our study, primarily because of the retrospective nature of the data analysis and the small sample size. These limitations highlight the need for future prospective studies to investigate the treatment of SND manifestations, such as bradyarrhythmias and asystolic cardiac arrest, particularly to elucidate the duration of temporary pacemaker use and the identification of criteria for their safe removal.

## Conclusions

Autonomic dysfunction associated with anti-NMDAR encephalitis can be life-threatening, significantly contributing to morbidity and mortality in severe cases. Clinicians must exercise extreme caution when prescribing negative chronotropic agents to patients with anti-NMDAR encephalitis, even those with inappropriate sinus tachycardia owing to the alternating nature of tachycardia and bradycardia and the potential for sinus pause or arrest. Our findings suggest that teratoma is more common in patients with SND and is associated with statistically nonsignificant less frequent use of permanent pacemakers. Although autonomic dysfunction is transient in most cases, approximately 26% of patients may experience a protracted course necessitating permanent pacemaker implantation. The exact vulnerable period for bradycardic and asystolic events is unknown, and there are no validated recommendations for the duration of temporary pacemaker use. The removal of temporary pacemakers should be individualized based on clinical improvement and the interpretation of pacemaker recordings to confirm the resolution of life-threatening asystolic and bradycardic events. In patients with severe disease and poor neurological status, it may be reasonable to consider extended periods of temporary pacemaker use, given the statistically nonsignificant odds ratio indicating a trend toward poor clinical outcomes in patients with permanent pacemakers. The important limitations of this review include the small sample size, which affects the statistical significance of the observed findings, and the retrospective nature of the study.
